# Complement Component 3 Negatively Regulates Antibody Response by Modulation of Red Blood Cell Antigen

**DOI:** 10.3389/fimmu.2018.00676

**Published:** 2018-06-11

**Authors:** Amanda Mener, Connie M. Arthur, Seema R. Patel, Jingchun Liu, Jeanne E. Hendrickson, Sean R. Stowell

**Affiliations:** ^1^Center for Transfusion Medicine and Cellular Therapies, Department of Pathology and Laboratory Medicine, Emory University School of Medicine, Atlanta, GA, United States; ^2^Department of Laboratory Medicine, Yale University School of Medicine, New Haven, CT, United States

**Keywords:** red blood cells, complement component 3, alloimmunization, antigen modulation, humoral immunity

## Abstract

Red blood cell (RBC) alloimmunization can make it difficult to procure compatible RBCs for future transfusion, directly leading to increased morbidity and mortality in transfusion-dependent patients. However, the factors that regulate RBC alloimmunization remain incompletely understood. As complement has been shown to serve as a key adjuvant in the development of antibody (Ab) responses against microbes, we examined the impact of complement on RBC alloimmunization. In contrast to the impact of complement component 3 (C3) in the development of an immune response following microbial exposure, transfusion of C3 knockout (C3 KO) recipients with RBCs expressing KEL (KEL RBCs) actually resulted in an enhanced anti-KEL Ab response. The impact of C3 appeared to be specific to KEL, as transfusion of RBCs bearing another model antigen, the chimeric HOD antigen (hen egg lysozyme, ovalbumin and Duffy), into C3 KO recipients failed to result in a similar increase in Ab formation. KEL RBCs experienced enhanced C3 deposition and loss of detectable target antigen over time when compared to HOD RBCs, suggesting that C3 may inhibit Ab formation by impacting the accessibility of the target KEL antigen. Loss of detectable KEL on the RBC surface did not reflect antigen masking by C3, but instead appeared to result from actual removal of the KEL antigen, as western blot analysis demonstrated complete loss of detectable KEL protein. Consistent with this, exposure of wild-type B6 or C3 KO recipients to KEL RBCs with reduced levels of detectable KEL antigen resulted in a significantly reduced anti-KEL Ab response. These results suggest that C3 possesses a unique ability to actually suppress Ab formation following transfusion by reducing the availability of the target antigen on the RBC surface.

## Introduction

Red blood cell (RBC) transfusion therapy can provide a life-saving intervention for patients with congenital hemoglobinopathies or general bone marrow failure syndromes (1–5). However, while RBC transfusion can be beneficial in a variety of patient populations, transfusion is not without risk. Patients who receive chronic transfusions are prone to developing alloantibodies against RBC alloantigens that differ between RBC donors and recipients (6, 7). Currently, the only method to reduce the development of alloantibodies against RBC antigens is to match donor and recipient for the common antigen targets of RBC alloimmunization. However, despite alloantigen matching protocols, 30–50% of chronically transfused patients can still become alloimmunized (4, 5, 8). The development of alloantibodies against various RBC antigens can make it difficult to find compatible blood for future transfusions (4, 8). Furthermore, individuals who develop alloantibodies are at an increased risk of developing hemolytic transfusion reactions (9–11), one of the most common causes of transfusion-related mortality (5, 12). As no current prophylactic measure exists that can actively inhibit RBC alloimmunization, a greater understanding of the molecular mechanisms that govern RBC alloantibody development is needed.

Previous studies demonstrate that in the absence of complement, little detectable humoral immunity can be observed following microbial challenge in mice (13–18). These results suggest that complement enhances the ability of hosts to drive an effective adaptive immune response, providing a key example of the intimate association between innate and adaptive immunity (14, 15). Previous studies also demonstrate that early alloantibodies directed against RBC alloantigens can fix complement (19–24), suggesting that complement fixation on the RBC surface may likewise favorably impact the development of alloantibodies against RBC alloantigens. However, in contrast to the well-established role of complement in the development of a humoral immune response to various microbes (13, 16, 18, 25, 26), the impact of complement fixation on the surface of transfused RBCs on the development of a humoral immune response to a RBC alloantigen remains incompletely understood. The lack of studies examining the consequence of complement deposition on RBCs following RBC transfusion on subsequent adaptive immunity, in part, reflects the fact that unlike solid organ transplantation, RBCs from different strains of mice do not inherently possess distinct antigenic differences known to routinely induce alloantibodies following transfusion between strains (27, 28). In contrast, alternative approaches employing RBCs from different species does result in an immune response. However, as xenograft transfusion of rat and guinea pig RBCs results in rapid RBC clearance and an inflammatory response that is likely not consistent with immune recognition observed between RBC donor and recipient clinically (29–31), models that more accurately recapitulate key features of RBC alloimmunization are needed.

In an effort to overcome limitations in the study of factors that regulate immune responses to distinct alloantigens on the surface of RBCs isolated from the same species, several murine models have been recently developed. Of all of these models, the KEL model system, which employs a β-globin promoter to drive expression of the clinically relevant human KEL antigen specifically on murine RBCs (KEL RBCs), currently provides the most compelling model capable of recapitulating key clinical features of RBC alloimmunization, including the ability to induce antibodies (Abs) capable of clearing transfused RBCs (32–35). Using this model system, we investigated the consequences of complement deposition following RBC transfusion. We found that in contrast to the impact of complement on the development of a humoral immune response to microbes (13–18), transfusion of KEL RBCs into complement component 3 knockout (C3 KO) recipients actually resulted in an increased Ab response to KEL. These results suggest that unlike microbes, C3 can actually play an inhibitory role in the development of Abs to transfused KEL RBCs and indicates a unique role for C3 in regulating adaptive immune responses directed against self.

## Materials and Methods

### Mice

Female C57BL/6 (B6) mice were purchased from National Cancer Institute (Bethesda, MD, USA). B6;129S4-C3tm1Crr/J (C3 KO) mice and C57BL/6N-*Hc^*tm1a(EUCOMM)Wtsi*^*/J (C5 KO) mice were purchased from Jackson Laboratories (Bar Harbor, ME, USA). Fcer1g mice, deficient in Fcγ receptors I, III, and IV (FcγR KO), were purchased from Taconic Laboratories (Hudson, NY, USA). Donor KEL and HOD transgenic mice, expressing only KEL or HOD on RBCs under the β-globin promoter, were generated as previously described (32, 33, 36), and were a generous gift from Dr. James Zimring (Bloodworks Northwest). All recipient and donor mice are on a C57BL/6 background and carry the H2^b^ haplotype. All mice were used at 8–12 weeks of age, and were bred and housed by Emory Animal Resources in accordance to policies outlined by the Institutional Animal Care and Use Committee. All experiments included three to five mice per group and were repeated at least three times.

### Mouse Genotyping and Screening

To confirm the genetic deletion of C3 in the C3 KO mice, DNA was isolated from peripheral blood using the DNeasy Blood and Tissue Kit (QIAGEN). Polymerase chain reaction was performed using Taq Polymerase Master Mix Red (Apex) with primer sequences provided by The Jackson Laboratory (common primer olMR1325 sequence: ATC TTG AGT GCA CCA AGC C, wild-type primer olMR1326 sequence: GGT TGC AGC AGT CTA TGA AGG, and mutant primer olMR7415 sequence: GCC AGA GGC CAC TTG TGT AG). To confirm the genetic deletion of activating Fcγ gamma receptors (FcγRs) in FcγR KO mice, peripheral blood was collected from B6 and FcγR KO mice into the anticoagulant acid citrate dextrose (ACD; Vacutainer, BD Bioscience), followed by RBC lysis 3× for 15 min each with 150 µL RBC Lysing Buffer Hybri-Max (Sigma, St. Louis, MO, USA). Following RBC lysis, peripheral lymphocytes were stained with anti-CD64 [(clone: X54-5/7.1), BioLegend, San Diego, CA, USA] diluted 1:100 in flow cytometry staining buffer, FACS buffer [0.1% BSA (bovine serum albumin) in phosphate buffered saline (PBS)], for 30 min at 4°C. To confirm KEL expression on KEL RBCs, peripheral blood was collected from KEL mice into ACD, washed 3× with FACS buffer and stained with anti-KEL Abs [(anti-Kp^b^, MIMA-9 and anti-Js^b^, MIMA-8), Bioxcell West Lebanon, NH, USA] diluted in FACS buffer 1:100 for 20 min at room temperature, as previously described (35), followed by incubation with anti-mouse IgG (Jackson Immunoresearch, West Grove, PA, USA) diluted 1:100 in FACS buffer for 20 min at room temperature. To confirm hen egg lysozyme (HEL) on HOD RBCs, peripheral blood was collected from HOD mice into ACD, washed 3× with FACS buffer and stained with anti-HEL monoclonal Abs [(clones: 2F4 and 4B7, both IgG1), Bioxcell, West Lebanon, NH, USA] diluted 1:100 in FACS buffer for 20 min at room temperature, followed by anti-mouse IgG (Jackson Immunoresearch, West Grove, PA, USA) diluted 1:100 in FACS buffer for 20 min at room temperature. RBC staining was measured by a FACSCalibur flow cytometer. Flow cytometric data were acquired by CellQuest Pro and analyzed using FlowJo software.

### Blood Collection and Transfusion

For alloimmunization experiments, KEL or HOD RBCs were collected into a 50-mL conical tube containing ACD and washed 3× in PBS. After buffy coat aspiration between washes, each mouse was transfused via the lateral tail vein with 50 µL packed KEL or HOD RBCs re-suspended in 300 µL PBS. To evaluate KEL or HOD RBC clearance, Ab deposition, antigen levels, and complement fixation at various time points post-transfusion, KEL, or HOD RBCs were collected into ACD and washed 3× in PBS. Following collection and washes, KEL or HOD RBCs were labeled with Molecular Probes Cell Tracker CM-DiI (1,1′-dioctadecyl-3,3,3′3′-tetramethylindocarbocyanine perchlorate; Life Technologies, Carlsbad, CA, USA) to enable differentiation of KEL or HOD RBCs from recipient RBCs post-transfusion. Control KEL or HOD negative RBCs (B6) were likewise labeled with a different lipophilic dye, DiO (3,3′-dihexadecyloxacarbocyanine perchlorate), to provide an internal KEL or HOD antigen-negative RBC control, as previously described (32, 34, 37, 38). Labeling was confirmed individually by a FACSCalibur flow cytometer prior to mixing and transfusion. Following washing 3× in PBS after labeling, DiI-KEL RBCs and DiO-KEL-negative RBCs were mixed equally. DiI-HOD RBCs and DiO-HOD-negative RBCs were also mixed equally. Each mouse was transfused with 50 µL packed DiI-KEL RBCs (1:1 with DiO-KEL-negative RBCs) or packed 50 µL DiI-HOD RBCs (1:1 with DiO-HOD-negative RBCs) re-suspended in 300 µL PBS into the lateral tail vein (32, 37, 38).

### Staining for Flow Cytometry

Following transfusion, peripheral blood was collected from each mouse into ACD and washed 3× in PBS. IgM and IgG on the RBC surface was detected through the direct antiglobulin test using anti-mouse IgM and IgG (Jackson Immunoresearch) diluted 1:100 in FACS buffer. Complement was detected using rat anti-mouse biotinylated Abs against an epitope within C3d (Cedarlane) or initial forms of C3b (C3b/iC3b) (Cedarlane), followed by streptavidin (BD). Then, peripheral blood was stained for the level of detectable KEL antigen using polyclonal anti-KEL Ab diluted 1:100 in FACS buffer and incubated for 20 min at room temperature, as done previously (33, 34, 37, 39). Peripheral blood was stained for the level of detectable HEL antigen using polyclonal anti-HEL Ab diluted 1:100 in FACS buffer for 20 min at room temperature (38). Stained RBCs were then washed 3× in FACS buffer and incubated with the secondary Ab anti-mouse IgG (Jackson Immunoresearch, West Grove, PA, USA) diluted 1:100 in FACS buffer for 20 min at room temperature, as done previously (33, 34, 37, 38, 40). For staining of white blood cells (WBCs), to evaluate the specificity of KEL and HOD expression, spleens were isolated from HOD and KEL donor mice. After washing in PBS and lysis with RBC Lysing Buffer (Sigma, St. Louis, MO, USA), WBCs were further washed 2× in PBS prior to staining, as done previously (41). For staining of platelets, HOD and KEL donor mice were exsanguinated into ACD and the peripheral blood was centrifuged at 80 *g* with 1:2 PBS to isolate platelet-rich plasma, as done previously (40, 42). WBCs, platelets, or RBCs were then stained with polyclonal anti-KEL Ab or polyclonal anti-HEL Ab diluted 1:100 in FACS buffer. Following washing in FACS buffer, cells were stained with anti-mouse IgG (Jackson Immunoresearch, West Grove, PA) diluted 1:100 in FACS buffer. After washing in FACS buffer, cells were stained with anti-CD45 (BD) for WBCs, anti-CD41 (BD) for platelets, or anti-Ter119 (BD) for RBCs. Stained RBCs were then washed 3× in FACS buffer and diluted to a final total volume of 100 µL in FACS buffer.

### Flow Cytometry

After staining, 50 µL of each set of stained cells in FACS buffer was then added to 400 µL of FACS buffer and the level of detectable antigen, Ab bound, or complement deposition was measured by a FACSCalibur flow cytometer by gating specifically on RBCs (Figure S1 in Supplementary Material). Data acquisition was accomplished by CellQuest Pro and was analyzed using FlowJo software (33, 34, 37). Mean fluorescence intensity (MFI) was used to assess the levels of detectable antigen, Ab bound, or complement deposition. For the level of detectable antigen, the MFI of experimental mice was expressed as a percentage of the MFI of DiI-KEL RBCs transfused into KEL mice or DiI-HOD RBCs transfused into HOD mice.

### Seroanalysis

To detect anti-KEL or anti-HOD alloantibody development in the serum, a flow cross-match was performed, as previously described (32, 33, 40, 41, 43). Briefly, 10 µL of serum was incubated for 15 min at room temperature with 3 µL of either KEL or HOD RBCs. RBCs were then washed 3× in FACS buffer, followed by incubation with anti-mouse IgM or IgG (Jackson Immunoresearch, West Grove, PA, USA) diluted 1:100 in FACS buffer for 30 min at room temperature. Non-specific background binding was accounted for through incubation of serum from alloimmunized mice with antigen-negative RBCs. Alloantibody development through binding of serum alloantibodies to the antigen-positive RBCs was measured by a FACSCalibur flow cytometry and analyzed using FlowJo software. Ab development was assessed in terms of MFI, as outlined previously (33, 40, 43). While the Abs detected following HOD or KEL RBC transfusion are not technically “alloantibodies,” they have been commonly referred to as alloantibodies in previous work, as the KEL and HOD systems are models of RBC alloimmunization (32, 33, 40, 43). In an effort to continue to provide uniformity of nomenclature within the field, we will continue to use this term to refer to Abs generated in response to KEL or HOD RBC transfusion in the present work.

### Western Blot Analysis

B6 mice were passively immunized with polyclonal anti-KEL Ab or injected with PBS, followed by transfusion with DiI-labeled KEL RBCs, as outlined previously (34). At day 1 post-transfusion, RBCs from immunized or non-immunized B6 mice were collected into ACD and washed 3× in PBS. Following washes, 100 µL of RBCs was incubated with 100 µL of protease inhibitor cocktail (Sigma) and 10 mL of RBC lysis buffer (5 mmoL/L sodium phosphate, pH 7.5). 1 mL aliquots of lysed RBCs were washed and centrifuged at 14,000 *g* for 10 min until RBC membranes were transparent, as done previously (43, 44). Following lysis and washes, membranes were re-suspended in 75 µL of 1× PBS with 25 µL of NuPage LDS Sample Buffer (4×) with 2.5% β-mercaptoethanol. Samples were heated at 70°C for 10 min then run on a reducing SDS-PAGE gel. Following transfer, the membrane was blocked in 5% low-fat milk, then incubated overnight at 4°C in primary, anti-KEL Ab (Abcam, clone: MM0435-12×3) diluted 1:250 in blocking buffer, as previously shown for detection of KEL (35, 39), or anti-GAPDH (Thermo Scientific, clone: GA1R) diluted 1:10,000 in blocking buffer for detection of GAPDH. Membranes were incubated in horseradish peroxidase-conjugated goat anti-mouse IgG1 (Bethyl Laboratories, Montgomery, TX, USA) diluted 1:1,000 in blocking buffer.

### Statistical Analysis

Flow cytometry data were analyzed by FlowJo software and statistical analyses were performed in GraphPad Prism. For comparisons between two groups, we utilized the unpaired Student’s *t*-test. For groups of three or more, we used one-way ANOVA analysis with multiple comparisons performed by Tukey’s post-test, unless otherwise noted. *p* < 0.05 was the cutoff for significance.

## Results

### Ab and C3 Specifically Deposit on KEL RBCs Post-Transfusion

**Figure 1:**
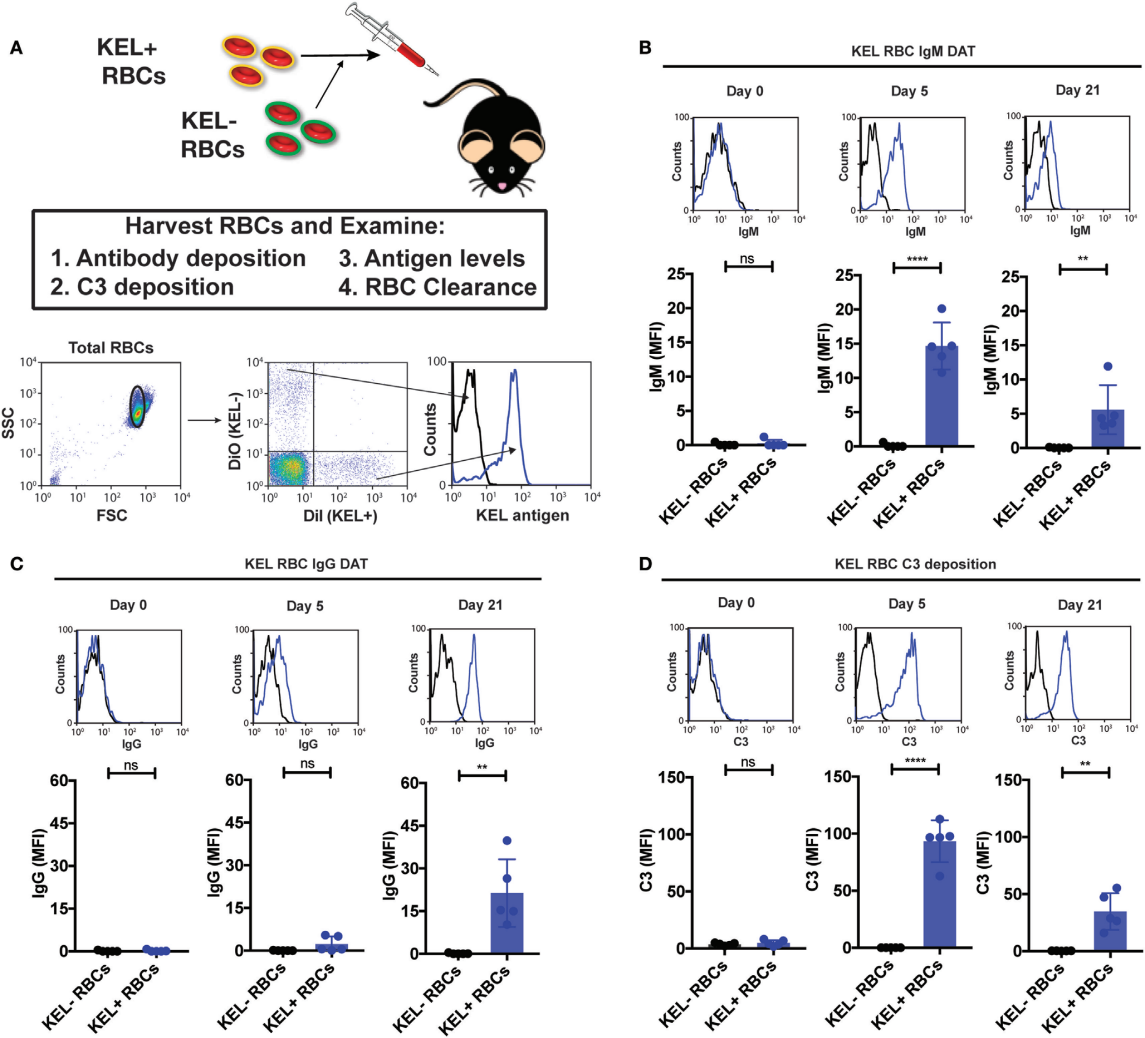
IgM, IgG, and complement component 3 (C3) specifically deposit on KEL positive red blood cells (RBCs) post-transfusion. **(A)** Schematic overview of approach used to detect IgM, IgG, and C3 on the surface of RBCs post-transfusion. Packed DiI-labeled KEL RBCs were co-transfused with packed DiO-labeled KEL negative B6 RBCs, followed by analysis of surface detectable IgM, IgG, C3, or antigen. Representative gating on DiI-labeled transfused KEL-positive RBCs or DiO-labeled KEL-negative RBCs with histogram analysis for the KEL antigen on each population. **(B)** KEL-negative RBCs (KEL− RBCs) or KEL positive RBCs (KEL+ RBCs) were evaluated at days 0, 5, and 21 post-transfusion for deposition of IgM on the surface of RBCs by flow cytometry in B6 mice. **(C)** KEL-negative RBCs (KEL− RBCs) or KEL-positive RBCs (KEL+ RBCs) were evaluated at days 0, 5, and 21 post-transfusion for deposition of IgG on the surface of RBCs by flow cytometry in B6 mice. **(D)** KEL-negative RBCs (KEL− RBCs) or KEL-positive RBCs (KEL+ RBCs) were evaluated at days 0, 5, and 21 post-transfusion for C3 deposition on the surface of RBCs by flow cytometry in B6 mice. **(B–D)** ****p < 0.0001, **p < 0.01 and ns = not significant. Means ± SD shown.

To define the impact of C3 on Ab formation following RBC transfusion, we first sought to determine whether Ab deposition and complement fixation occurs following transfusion of KEL RBCs. To accomplish this, we first labeled packed KEL RBCs with a lipophilic dye, DiI, prior to transfusion to facilitate flow cytometric detection at various time points post-transfusion (Figure 1A). To determine the specificity of potential Ab and complement interactions with KEL RBCs, RBCs that do not express KEL were labeled with a fluorescently distinct lipophilic dye, DiO, followed by co-transfusion with KEL RBCs into each recipient (Figure 1A). This approach allows specific detection of each RBC population as a distinct population with the predicted antigen expression following transfusion (Figure 1A; Figures S1 and S2 in Supplementary Material). While no Ab could initially be detected on the surface of transfused KEL RBCs, by day 5, statistically significant IgM could be observed on the surface of KEL RBCs (Figure 1B). These Abs gradually increased over time and switched from primarily IgM deposition to IgG by day 21 post-transfusion (Figure 1C), suggesting that the Abs that form in response to KEL RBC transfusion possess the capacity to specifically engage the KEL RBC target *in vivo* during the developing immune response. Ab engagement appeared to be specific to KEL RBCs, as KEL negative RBCs circulating in the same recipients failed to exhibit significant Ab deposition (Figures 1B,C). Together, these data indicate that transfusion of KEL RBCs appears to result in formation of anti-KEL Abs that can specifically engage KEL RBCs.

Given the ability of Abs to specifically engage KEL RBCs (Figure 1), we next determined whether Ab engagement results in the deposition of complement during the developing immune response. Previous studies have demonstrated that Ab-mediated complement deposition first occurs as a cleavage product of C3 to C3b, which covalently attaches to the cell surface, but can be quickly degraded into the complement split product, C3d, that remains covalently attached to the cell surface (45–48); C3d is the common covalently attached complement target evaluated clinically when complement-mediated processes on the RBC surface are suspected (24). As a result, we not only examined early complement deposition following Ab engagement, but also determined the relative amount of early versus degraded C3 on KEL RBCs over time. As no anti-C3 Ab is currently available that can specifically differentiate C3d from total C3 (given that C3d is part of the entire C3 protein), early versus degraded complement detection can be accomplished by examining cells for epitopes of C3b and iC3b that are removed following degradation to C3d and comparing this to total C3 using an Ab that recognizes an epitope within C3d (34). Following transfusion, total C3 could be readily detected on the surface of KEL RBCs (Figure 1D). In contrast, very little C3b could be detected on the cell surface at any time point evaluated (Figures S3A–D in Supplementary Material), suggesting that complement activated on the KEL RBC surface during the development of an immune response rapidly degrades to C3d. No C3 could be detected on the surface of KEL negative RBCs co-transfused with KEL RBCs (Figure 1D), suggesting that anti-KEL Ab engagement specifically occurred on KEL RBCs and that this appears to in turn result in KEL RBC-specific C3 deposition. Taken together, these results demonstrate that Abs that form in response to KEL RBC transfusion not only possess the ability to specifically engage KEL RBCs but also can fix complement.

### C3 KO Recipients Exhibit an Increased Immune Response to Transfused KEL RBCs

**Figure 2:**
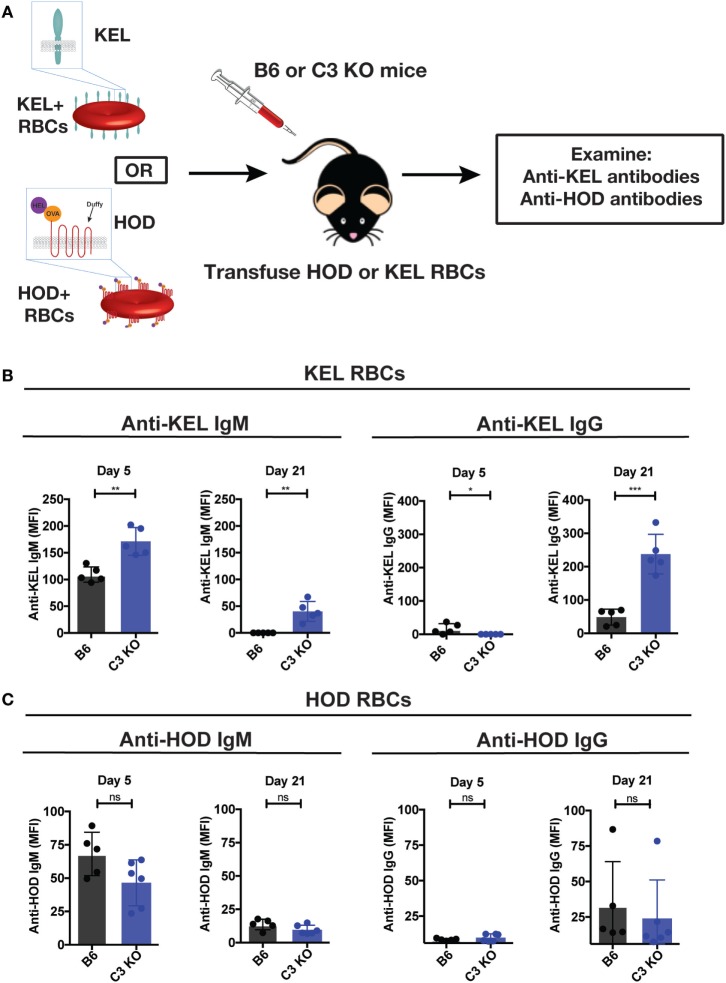
Complement component 3 (C3) KO recipients exhibit an increased anti-KEL antibody (Ab) response following KEL red blood cell (RBC) transfusion. **(A)** Schematic overview of KEL or HOD RBC transfusion into B6 and C3 KO mice, followed by examination of anti-KEL or anti-HOD Abs, respectively. **(B)** Following exposure to KEL RBCs, serum from B6 or C3 KO recipients was assessed for development of anti-KEL IgM and IgG at days 5 and 21, respectively, following transfusion by flow cross-match. **(C)** Following exposure to HOD RBCs, serum from B6 or C3 KO recipients exposed to HOD RBCs was assessed for development of anti-HOD IgM and IgG at days 5 and 21, respectively, post-transfusion by flow cross-match. **(B,C)** ***p < 0.0002, **p < 0.004, *p < 0.05 and ns = not significant. Means ± SD shown.

Given the impact of C3 on developing immune responses following microbial challenge and the ability of Abs that develop in response to KEL RBC transfusion to fix complement (13–18), we next sought to directly examine the potential impact of complement on the immune response following KEL RBC transfusion. To accomplish this, we transfused B6 or C3 KO recipients with KEL RBCs, followed by an evaluation of anti-KEL Ab formation over time (Figure 2A). While KEL RBCs induced anti-KEL Abs in B6 recipients, as seen previously (32, 34, 44), similar exposure in C3 KO recipients unexpectedly resulted in a statistically significant increase in IgM anti-KEL Ab formation by day 5 post-exposure (Figure 2B). Similar increases in IgG anti-KEL Abs were also observed in C3 KO recipients at day 21 post-exposure (Figure 2B). To determine whether the increased Ab response observed in C3 KO recipients was specific to KEL RBCs, we next determined whether RBCs expressing an entirely different model RBC alloantigen, the HOD (HEL, ovalbumin and human Duffy) antigen, likewise induced an enhanced immune response following transfusion into C3 KO recipients. In contrast to the increased anti-KEL Ab response observed following transfusion of KEL RBCs into C3 KO recipients (Figure 2B), transfusion of C3 KO recipients with HOD RBCs failed to result in increased anti-HOD IgM or IgG (Figure 2C). The ability of KEL RBCs to induce an enhanced anti-KEL Ab response stands in stark contrast to previous studies implicating a key requirement for C3 in the development of Abs following pathogen exposure (13, 18, 25, 49–53). Instead, these results suggest that C3 may actually play an inhibitory role when Abs develop against the KEL RBC alloantigen following KEL RBC transfusion.

### Complement Facilitates Loss of Detectable KEL Antigen Independent of Changes in RBC Clearance

**Figure 3:**
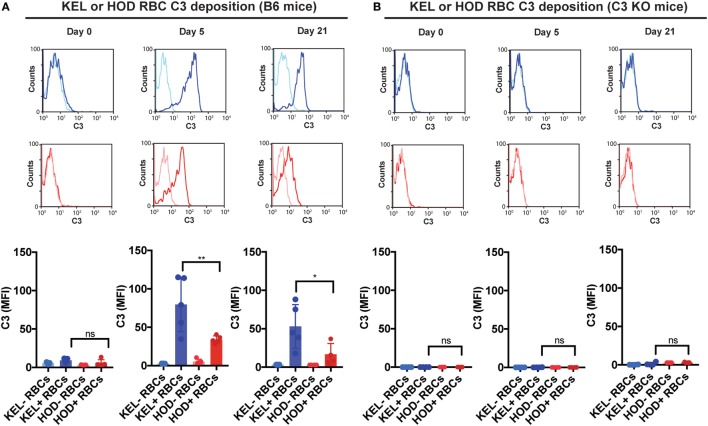
KEL red blood cells (RBCs) exhibit increased levels of complement component 3 (C3) deposition when compared to HOD RBCs over time post-transfusion. **(A)** Total C3 bound to circulating KEL-negative RBCs (KEL− RBCs), KEL-positive RBCs (KEL+ RBCs), HOD-negative RBCs (HOD− RBCs) or HOD-positive RBCs (HOD+ RBCs) was assessed on days 0, 5, and 21 post-transfusion into B6 mice. **(B)** Total C3 bound to circulating KEL-negative RBCs (KEL− RBCs), KEL-positive RBCs (KEL+ RBCs), HOD-negative RBCs (HOD− RBCs) or HOD-positive RBCs (HOD+ RBCs) was assessed on days 0, 5, and 21 post-transfusion into C3 KO mice. **(A,B)** **p < 0.005, *p < 0.05 and ns = not significant. Means ± SD shown.

**Figure 4:**
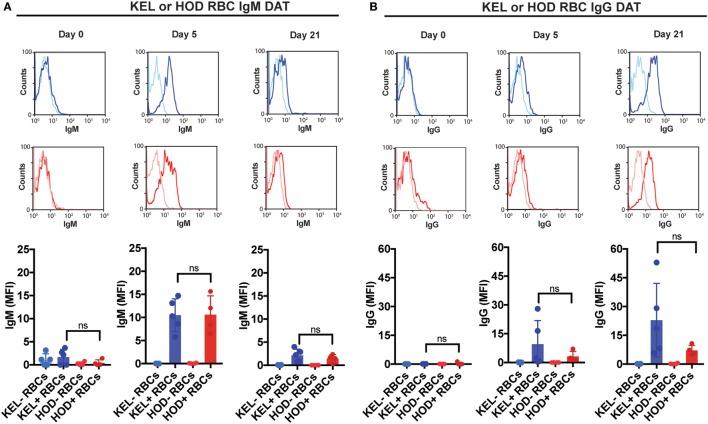
IgM and IgG specifically deposit on KEL or HOD red blood cells (RBCs) at similar levels post-transfusion. **(A)** KEL-negative RBCs (KEL− RBCs), KEL-positive RBCs (KEL+ RBCs), HOD-negative RBCs (HOD− RBCs) or HOD-positive RBCs (HOD+ RBCs) were evaluated at days 0, 5, and 21 post-transfusion for surface engagement of IgM by flow cytometry in B6 mice. **(B)** KEL-negative RBCs (KEL− RBCs), KEL-positive RBCs (KEL+ RBCs), HOD-negative RBCs (HOD− RBCs) or HOD-positive RBCs (HOD+ RBCs) were evaluated at days 0, 5, and 21 post-transfusion for surface engagement of IgG by flow cytometry in B6 mice. **(A,B)** ns = not significant. Means ± SD shown.

Given the impact of C3 on anti-KEL Ab formation specifically, we first sought to determine whether differences in C3 deposition on the RBC surface might correlate with differences in Ab formation observed following KEL or HOD RBC transfusion into B6 versus C3 KO recipients. This is especially important when considering that previous studies suggest that Ab engagement of different RBC antigens can differentially impact the likelihood of complement activation (24). While total C3 could be readily detected specifically on the surface of KEL RBCs, significantly less complement could be detected on HOD RBCs when evaluated in parallel (Figure 3A). Importantly, transfusion of HOD or KEL RBCs into C3 KO recipients failed to result in detectable C3 deposition (Figure 3B), which demonstrated that the detection of C3 was likely specific. Furthermore, differences in C3 deposition did not appear to reflect alterations in the level of initial Ab engagement, as the level of detectable Ab on the surface of KEL RBCs or HOD RBCs appeared to be very similar (Figure 4). While a trend toward increased IgG1, IgG2b, and IgG3 Abs following KEL RBC transfusion was observed, compared to HOD RBC-induced Ab formation, these differences failed to reach statistical significance (Figure S3 in Supplementary Material). Taken together, these results demonstrate that Abs that form in response to both KEL and HOD RBC transfusion can fix complement. However, differences in the level of C3 deposition on KEL and HOD RBCs may impact the ability of C3 to regulate alloantibody formation specifically following KEL RBC transfusion.

**Figure 5:**
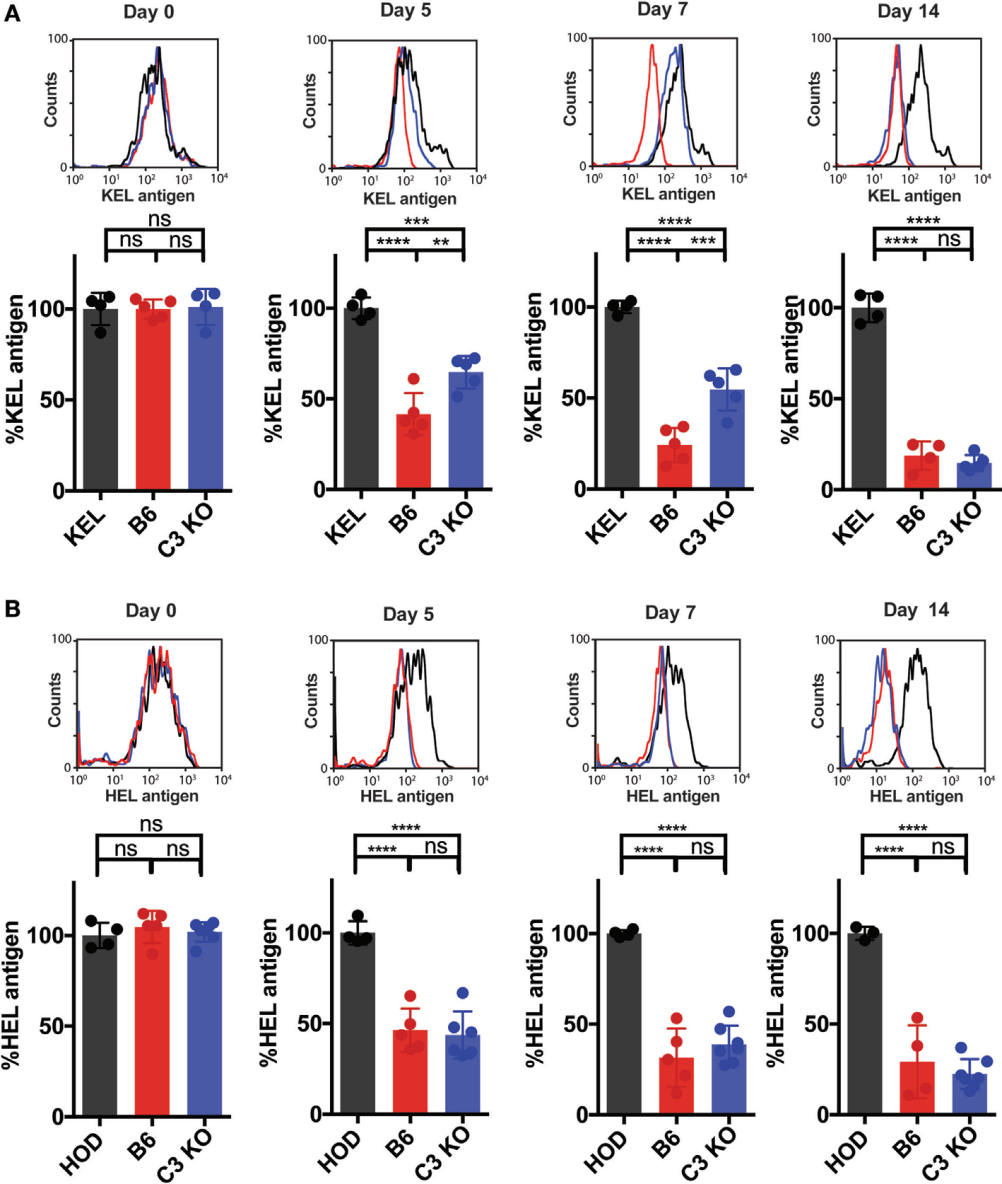
Complement component 3 (C3) accelerates loss of detectable KEL antigen on KEL red blood cells (RBCs) over time. **(A,B)** B6 or C3 KO recipients were transfused with either DiI-labeled KEL RBCs or DiI-labeled HOD RBCs with control DiO-labeled antigen-negative RBCs. As a control, KEL mice were transfused with syngeneic DiI-labeled KEL RBCs or HOD mice were transfused with syngeneic DiI-labeled HOD RBCs, each with control DiO-labeled antigen-negative RBCs. **(A)** Post-transfusion, DiI-labeled KEL RBCs were stained for the level of detectable KEL antigen using anti-KEL polyclonal antibody (Ab). Level of detectable KEL antigen was measured as a percentage of KEL antigen normalized to the level of detectable KEL antigen in KEL mice transfused with syngeneic DiI-KEL RBCs, shown at days 0, 5, 7, and 14 post-transfusion. **(B)** Post-transfusion, DiI-labeled HOD RBCs were stained for the level of detectable hen egg lysozyme (HEL) antigen using polyclonal anti-HEL Ab. Level of detectable HEL antigen was measured as a percentage of HEL antigen normalized to the level of detectable HEL antigen in HOD mice transfused with syngeneic DiI-HOD RBCs. **(A,B)** ****p < 0.0001, ***p < 0.0008, **p < 0.007 and ns = not significant. Means ± SD shown.

The differential complement deposition on the RBC surface of KEL and HOD RBCs, coupled with previous studies suggesting that complement may potentially impact the availability of the target antigen (54), suggests that complement may physically mask or otherwise alter the availability of the KEL antigen to the ongoing immune response. This in turn would be predicted to impact the ongoing anti-KEL immune response. To test this, we next determined the consequence of Ab engagement and C3 deposition on KEL antigen availability on the KEL RBC surface over time. As a control, we also transfused KEL RBCs into KEL donor mice, which do not generate anti-KEL Abs (32). This allows parallel evaluation of the overall stability of the KEL antigen over time following transfusion in the absence of an immune response. While the KEL antigen could be readily detected initially following transfusion into KEL, B6, or C3 KO mice, consistent with the possibility that C3 may actually regulate KEL antigen accessibility during the developing immune response, transfusion of KEL RBCs into B6 recipients actually resulted in decreased levels of detectable KEL antigen over time when compared to control KEL mice transfused with syngeneic KEL RBCs (Figure 5A). This decrease in the level of detectable KEL antigen in B6 recipients also correlated with the development of anti-KEL Abs (Figure 2B). In contrast, a similar decrease in the level of detectable KEL antigen over time failed to occur at the same rate in C3 KO recipients, suggesting that while Ab itself may limit accessibility to the KEL antigen, C3 clearly accelerates this process (Figure 5A). To determine the specificity of C3-dependent changes to the KEL antigen over time, given the inability of C3 to regulate HOD RBC-induced Ab formation, we next evaluated the potential impact of C3 on the HOD antigen following transfusion. In contrast to KEL RBCs, alterations in the levels of detectable HEL antigen over time did not differ following transfusion into B6 or C3 KO mice (Figure 5B). Taken together, these results suggest that C3 may negatively regulate immunity toward KEL by impacting the availability of the KEL antigen on the KEL RBC surface.

**Figure 6:**
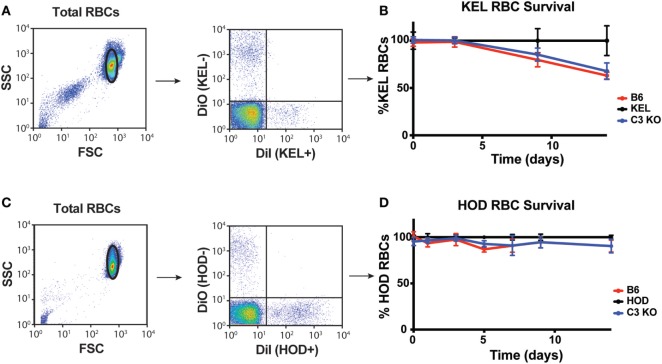
Complement component 3 (C3) fails to induce detectable alterations in KEL or HOD red blood cell (RBC) clearance post-transfusion. **(A)** Gating strategy of KEL+ or KEL− RBCs post-transfusion, where the percentage of KEL+ RBCs was directly compared to KEL− RBCs in each recipient. **(B)** KEL+ RBC survival was calculated as a ratio of KEL+ RBC to KEL− RBC in KEL mice, B6 or C3 KO recipients at the times indicated. **(C)** Gating strategy of HOD+ or HOD− RBCs post-transfusion, where the percentage of HOD+ RBCs was directly compared to HOD− RBCs in each recipient. **(D)** HOD+ RBC survival was calculated as a ratio of HOD+ RBC to HOD− RBC in HOD mice, B6 or C3 KO recipients at the times indicated. **(B,D)** RBC clearance was not significant between B6 and C3 KO recipients for KEL RBC **(B)** or HOD RBC **(D)** clearance by two-way ANOVA with Dunnett’s multiple comparisons test. Means ± SD shown.

To control for the possibility that complement may accelerate RBC removal and, therefore, impact KEL RBC immunogenicity independent of C3-induced alterations in the levels of detectable antigen on the RBC surface, we next sought to determine whether C3 impacts KEL RBC removal during the developing anti-KEL immune response. To accomplish this, labeled DiI-KEL RBCs were transfused into B6 and C3 KO recipients and the relative rate of KEL RBC removal compared to DiO-KEL negative RBCs co-transfused with KEL RBCs was determined over time (Figure 6A). Consistent with the lack of detectable active complement on the cell surface (Figures S4A–D in Supplementary Material) (24, 34, 55, 56), no increase in KEL RBC clearance was observed in B6 mice as compared to C3 KO recipients (Figure 6B), suggesting that C3 does not appear to induce detectable increases in the clearance of KEL RBCs following transfusion. Similarly, no differences in HOD RBC clearance were observed between B6 and C3 KO recipients (Figures 6C,D). These results suggest that while C3 can be deposited on the RBC surface, it does not appear to impact KEL or HOD RBC removal.

**Figure 7:**
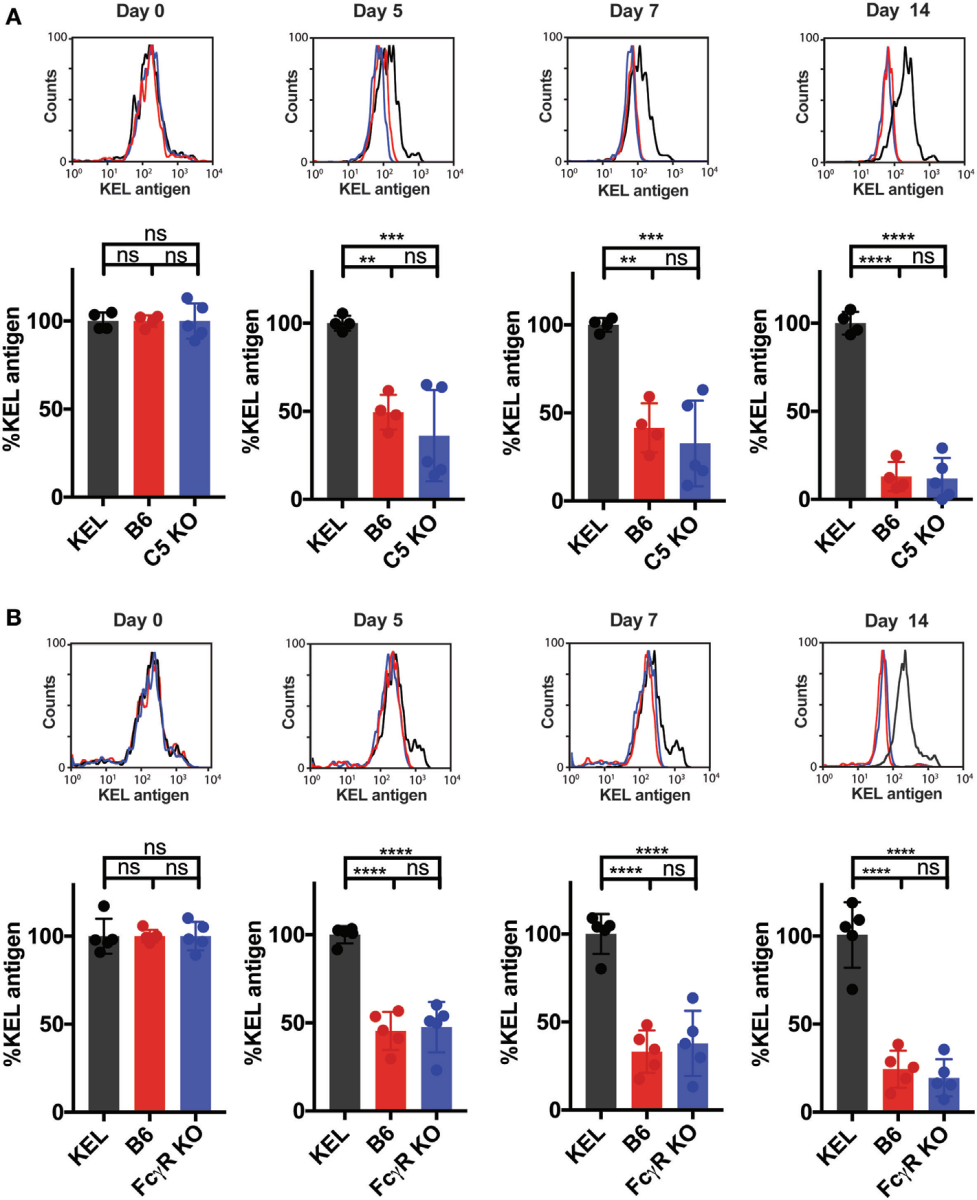
C5 and activating Fcγ gamma receptors (FcγRs) do not impact KEL antigen levels on transfused KEL red blood cells (RBCs) over time. **(A,B)** B6, C5 KO, and FcγR KO mice were transfused with DiI-labeled KEL+ RBCs and DiO-labeled KEL− RBCs. As a control, KEL mice were transfused with syngeneic DiI-labeled KEL+ RBCs and DiO-labeled KEL− RBCs. Post-transfusion, DiI-labeled KEL+ RBCs were stained for level of detectable KEL antigen using an anti-KEL polyclonal antibody. The level of detectable KEL antigen was measured as a percentage of KEL antigen normalized to the level of detectable KEL antigen in KEL mice transfused with syngeneic DiI-KEL+ RBCs, shown at days 0, 5, 7, and 14 post-transfusion for either C5 KO **(A)** or FcγR KO **(B)** mice. **(A,B)** ****p < 0.0001, ***p < 0.0007, **p < 0.006 and ns = not significant. Means ± SD shown.

**Figure 8:**
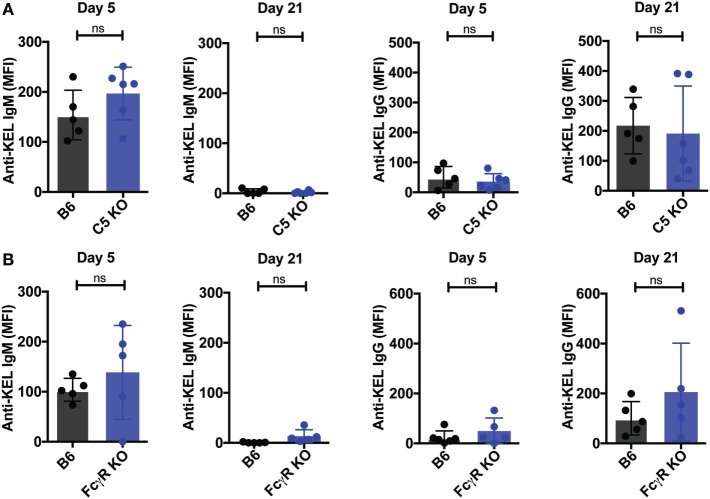
C5 KO and Fcγ gamma receptor (FcγR) KO mice exhibit no increase in the anti-KEL antibody response following KEL RBC transfusion. **(A,B)** B6, C5 KO, or FcγR KO mice were transfused with KEL RBCs. Anti-KEL IgM and IgG was measured at days 5 and 21 in the serum of C5 KO **(A)** or FcγR KO mice **(B)**. **(A,B)** ns = not significant. Means ± SD shown.

To assess whether other immune factors, independent of C3, may regulate KEL antigen availability on KEL RBCs post-transfusion over time, we examined the potential impact of C5, a downstream complement effector from C3 (57). Transfused KEL RBCs in C5 KO recipients were then assessed for the level of detectable KEL antigen compared to KEL RBCs transfused into syngeneic KEL RBC recipients. In contrast to the impact of C3 on the accessibility of the KEL antigen on KEL RBCs, transfusion of KEL RBCs into C5 KO mice resulted in a similar decrease in detectable KEL antigen levels when compared to B6 mice (Figure 7A), suggesting that the downstream complement effector, C5, is not required for alterations in detectable KEL antigen levels. As previous studies suggest that FcγR may also possess the ability to impact antigen accessibility (37, 38, 58), we next examined the potential impact of activating FcγRs on KEL antigen availability using the common gamma chain KO mouse (FcγR KO), as done previously (37, 38). However, similar to C5 KO recipients, no difference in KEL antigen could be detected when comparing KEL RBCs following transfusion into B6 or FcγR KO recipients (Figure 7B). Given the lack of alterations in KEL antigen observed in C5 KO or FcγR KO recipients, we next examined whether transfusion of KEL RBCs into C5 KO or FcγR KO recipients impacts the anti-KEL Ab response. Transfusion of KEL RBCs into C5 KO and FcγR KO mice failed to result in altered levels of anti-KEL IgM or IgG when compared to B6 mice (Figures 8A,B), suggesting that while C3 plays an inhibitory role in the Ab response to KEL, C5 and FcγRs do not appear to negatively or positively impact antigen levels or anti-KEL Ab formation following KEL RBC transfusion.

### Ab-Induced Antigen Changes on the RBC Surface Impact Ab Response

**Figure 9:**
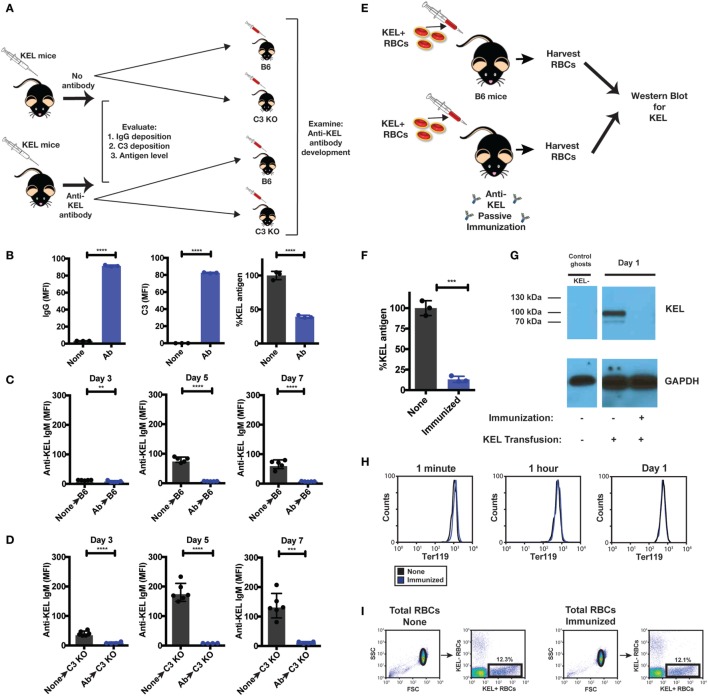
Re-transfusion of anti-KEL polyclonal antibody (Ab) treated KEL red blood cells (RBCs) into B6 or complement component 3 (C3) KO mice results in reduced anti-KEL Ab formation. **(A)** Schematic overview: anti-KEL polyclonal Ab or phosphate-buffered saline (PBS) (none) was injected into KEL donors, followed by initial evaluation for IgG deposition, C3 deposition, and KEL antigen levels. These isolated RBCs were then retransfused into B6 or C3 KO recipients, which were subsequently evaluated for anti-KEL Ab formation. **(B)** Following injection, IgG, C3, and detectable KEL antigen were assessed on transfused KEL RBCs in KEL donors. **(C,D)** KEL RBCs from PBS (none) or anti-KEL Ab-treated (Ab) KEL donors were harvested, followed by transfusion into KEL-negative naïve B6 **(C)** or C3 KO recipients **(D)** and assessed for anti-KEL IgM Abs on days 3, 5, and 7 post-transfusion. **(E)** Schematic overview: KEL-negative B6 mice were exposed to KEL RBCs in the absence (none) or presence of polyclonal anti-KEL Ab (immunized) at a volume adjusted ratio to ensure equivalent KEL RBCs were present in each group 1 day following transfusion. Day 1 post-transfusion, the level of detectable KEL antigen was measured by flow cytometry and western blot analysis. **(F,G)** Day 1 post-transfusion, the level of detectable KEL antigen was measured by flow cytometry **(F)** or western blot analysis **(G)**. **(H)** The level of KEL RBC Ter119 expression was assessed specifically on DiI-labeled transfused KEL positive RBCs at 1 min, 1 h, and Day 1 post-transfusion in immunized and non-immunized B6 mice by flow cytometry. **(I)** Gating strategy shows KEL RBC population in the absence (None) or presence of polyclonal anti-KEL Ab (Immunized). **(B–F)** **p < 0.009, ***p < 0.0004 and ****p < 0.0001. Means ± SD shown.

The results thus far suggest that Ab-induced deposition of complement on KEL RBCs impacts the availability of the cell surface KEL antigen, which in turn may reduce immune detection and, therefore, the magnitude of the ongoing anti-KEL immune response. In order to directly examine the consequence of alterations in the levels of detectable KEL antigen on the development of an anti-KEL immune response, we next sought to evaluate the impact of reduced KEL antigen availability on the development of anti-KEL Abs following KEL RBC transfusion. To accomplish this, we first induced alterations to the KEL antigen on KEL RBCs in KEL donors prior to RBC isolation and transfusion into separate recipients by directly injecting anti-KEL Abs into KEL RBC donors (Figure 9A). Injection of anti-KEL Abs in this manner resulted in rapid Ab engagement and complement deposition on KEL RBCs (Figure 9B). The level of detectable KEL antigen likewise decreased to approximately 50% of the initial values (Figure 9B), providing a unique KEL RBC substrate to directly test the impact of reduced KEL availability on the ability of KEL RBCs to induce Ab formation. As a result, we next transferred KEL RBCs from anti-KEL Ab treated or non-treated KEL donors into B6 or C3 KO recipients. Consistent with the possibility that reduced KEL antigen availability may impact anti-KEL Ab formation, B6 and C3 KO recipients that received KEL RBCs with decreased KEL antigen failed to develop a significant anti-KEL Ab response in either B6 or C3 KO mice (Figures 9C,D), while unaltered KEL RBCs induced a robust Ab response when evaluated in parallel. These results suggest that reductions in the level of detectable KEL on the KEL RBC surface can significantly impact the development of an anti-KEL Ab response.

Next, we sought to determine whether loss of detectable KEL antigen reflects masking of the KEL antigen or removal from the KEL RBC surface following allogeneic KEL RBC transfusion. To accomplish this, we injected B6 mice with polyclonal anti-KEL Ab prior to exposure to KEL RBCs, followed by RBC harvest 24 h following KEL RBC transfusion into these immunized or non-immunized recipients (Figure 9E). KEL RBCs were then evaluated for the level of detectable KEL antigen on transfused KEL RBCs first by flow cytometry. Transfusion of KEL RBCs into immunized recipients resulted in a near to complete loss of detectable KEL antigen, as detected by flow cytometry 24 h following injection (Figure 9F). To determine whether loss of KEL antigen in this setting reflected loss or simple masking of KEL, KEL RBCs transfused into immunized or non-immunized recipients were then subjected to western blot analysis. While KEL could be readily detected in B6 recipients injected with KEL RBCs in the absence of anti-KEL Abs (non-immunized), no KEL could be detected in anti-KEL immunized recipients (Figure 9G). These results suggest that the loss of detectable KEL, as measured by flow cytometry, likely reflects actual removal of the KEL antigen from KEL RBCs. In each of these situations, the loss of KEL antigen appeared to be specific, as similar changes in the RBC-specific cell surface marker, Ter119, failed to similarly occur (Figure 9H). Importantly, loss of detectable KEL antigen on transfused KEL RBCs was not due to significant KEL RBC clearance and, therefore, a simple reduction in KEL RBC numbers, as KEL RBCs were transfused at a pre-adjusted ratio to ensure that the final KEL RBC percentage at the time of western blot analysis were the same (Figure 9I). These results suggest that complement appears to facilitate removal of the KEL antigen, thereby reducing the availability of KEL for the ongoing anti-KEL immune response, while also leaving the remaining KEL RBCs intact.

## Discussion

The ability of C3 to negatively impact the anti-KEL Ab response reveals an unexpected role for C3 in regulating Ab formation against an antigen expressed on transfused RBCs. Previous studies demonstrate a critical role for C3 in the development of Abs toward a wide variety of microbes (13–18). Unlike microbes, RBCs express key complement-regulatory proteins, including CD55 and CD59, which regulate complement activation by interfering with complement assembly and effector function (59). While the exact nature of complement regulation can vary between species, the general features that govern complement activation that evolved to protect self from complement are highly conserved and represent an evolutionarily ancient process (24, 60, 61). The results of the present study suggest that in addition to the differences in the outcome of complement activation on the surface of a RBC when compared to a microbe (24, 34), which can protect RBCs from complement-mediated injury and removal in the presence or absence of Abs (34, 54, 62–64), complement also appears to possess the ability to negatively regulate the immune response toward an antigen on a RBC surface by impacting the availability of the target antigen for the ongoing immune response. Given the complexity of complement inactivation on the cell surface, in addition to many different complement receptors (CRs) and cell populations that can engage C3 (65–68), the regulation of C3-mediated target antigen removal on the RBC surface may also reflect an equally complex and coordinated process. This process may have co-evolved with complement activation of adaptive immunity to actually protect hosts from undesirable immune responses once directed toward an antigen on a self-like surface, such as a transfused RBC. Thus, while protection of cells from complement effector function represents a well-documented, evolutionarily conserved process with significant implications in human disease (62–64, 69, 70), the ability of complement to likewise negatively regulate Ab formation following engagement of RBCs provides a previously unrecognized additional ability of complement to differentially regulate adaptive immunity.

The ability of Abs in general to suppress immune function represents a long recognized, yet poorly understood process previously suggested to reflect a key regulatory loop in preventing or reducing Ab formation (71, 72). While previous studies have associated Ab-mediated immunosuppression with complete removal of antigen-positive cells, the development of anti-idiotype Abs and a variety of other mechanisms (21, 73), the ability of complement to negatively regulate Ab formation specifically following KEL RBC transfusion represents a unique role of complement in the regulation of Ab formation. This is especially important when considering that unlike cellular immunity, where intimate contact between effector cells and host targets allows hosts to directly regulate immune activity (74, 75), once Abs are released from Ab secreting cells, the target tissue is often spatially and temporally separated from the Ab secreting cell, making it difficult, if not impossible, for the target tissue to provide direct feedback to specific Ab secreting cells (76, 77). These data suggest that, along with the expression of complement-regulatory proteins on RBCs to protect against complement effector functions (34, 54, 62–64), RBCs may have also evolved distinct mechanisms to directly regulate the impact of Ab binding in order to favorably inhibit the consequence of an undesirable immune response when complement deposition does occur. As this process fails to occur following HOD RBC transfusion, these results also suggest that a threshold of C3 activation may be required for efficient C3-mediated regulation of an ongoing immune response. Thus, only when sufficient complement activation occurs does complement appear to be able to impact alloantibody formation. As recent studies suggest that RBCs with decreased levels of antigen may not possess the ability to induce Abs despite equivalent levels of total antigen exposure (41), the presentation of antigen on the cell surface appears to be critical for effective immune recognition and response. Thus, C3-mediated antigen loss, even if not initially complete, may reduce KEL antigen levels below a threshold required to efficiently contribute to an ongoing immune response. The ability of complement to accelerate actual removal of the target antigen, while leaving the transfused RBCs intact, may, therefore, provide an additional layer of host protection against unwanted immunity.

Our results are consistent with previous studies and demonstrate that Ab engagement of RBC antigens does not uniformily result in similar levels of complement fixation (24, 29, 30, 39, 78). Characteristics of the RBC-bound Ab, including potential differences in IgG subclass levels, as well as inherent biochemical differences between RBC antigens themselves may influence the ability of Ab to preferentially induce complement fixation following antigen engagement. This may be especially apparent when considering that while a single IgM molecule can initiate complement deposition through engagement with C1q, two molecules of IgG must be bound in close proximity to similarly engage C1q (24, 29, 30, 79), suggesting that differences in antigen density, lateral mobility and sites of Ab-antigen engagement may impact the relative ability of IgM and/or IgG Abs to efficiently initiate complement activation following Ab binding (24, 80). The increased ability of IgM to activate complement may be particularly important in the setting of KEL and HOD RBC transfusion when considering that the maximum complement deposition on transfused KEL or HOD RBCs occurred 5 days post-transfusion when IgM anti-KEL or anti-HOD levels peak and virtually no detectable corresponding IgG anti-KEL or anti-HOD Abs were present. Indeed, these results suggest that while IgG subclass could certainly impact predilections for C3 fixation following Ab-antigen engagement, unique antigen characteristics that differ between KEL and HOD may in part regulate the ability of IgM anti-KEL to more efficiently fix C3 on the RBC surface. This is especially important when considering that in contrast to the KEL antigen, which is a single membrane pass antigen and, therefore, may possess greater lateral mobility within the RBC membrane (81), the HOD RBC antigen contains the seven transmembrane pass human antigen, Duffy (36, 82), which may not lend itself to the same level of optimal IgM engagement required for efficient C1q binding and subsequent C3 fixation. Although many studies in the past have recognized that certain Ab-antigen combinations differentially fix complement (24, 29, 30, 39), future studies will certainly be needed to determine the underlying mechanisms that dictate whether C3 fixation will occur following Ab engagement of a particular RBC antigen.

Given the evolutionary ancient role of C3 in providing direct immunity, in addition to regulating immune function (83), many different cell types interact with C3 through a variety of CRs (84). As RBCs pass through many organs, including splenic sinusoids, cells such as red pulp macrophages may phagocytose RBCs following Ab engagement and complement fixation (85, 86). Additional cells, such as other CR-bearing myeloid cells in the spleen, blood, or other compartments, may likewise participate or primarily be responsible for this process (87). Given that there are many different immune populations that express various CRs, the processes that govern C3-mediated RBC clearance and antigen removal are also likely complex, and may involve multiple cell types and CRs. For example, while the CR of the immunoglobulin family (CRIg) is a more recently described CR, CRIg may work in concert with the more classically described CRs 1 through 4 (65, 67, 68, 88), suggesting a cooperative role of CR function. This is especially important when considering that RBCs transverse the spleen and other vascular tissue, where a variety of CR-bearing immune populations reside, each of which can express distinct CRs and have been previously shown to facilitate immune complex removal (89–91). While CRs can engage C3, they often display distinct preferences for various forms of C3 following activation (84, 92). Although bound forms of C3, such as C3b and iC3b, could not be detected on the surface of KEL RBCs, Ab-induced complement activation at the cell surface would be predicted to initially produce C3b, followed by iC3b, even if only transiently, which may in turn facilitate interactions between KEL RBCs and different CRs as KEL RBCs continue to circulate following Ab engagement (68, 93). As C5 did not appear to impact antigen levels or Ab formation, these results suggest that C5 or corresponding C5 receptors are not required for this process to occur. Although C3 and C5 are often the dominate players at key junctures in Ab-mediated complement activation, both with respect to direct activation of downstream complement pathways and the engagement of receptors capable of mediating complement responses (57), these results do not rule out a potential role for C4 in this process. Finally, although FcγRs do not appear to be required for antigen loss in the setting of KEL RBC transfusion, they have been shown to be involved in the induction of alterations to target antigens in other settings (37, 58), suggesting that Ab engagement of different Ab effector systems may, in general, possess the capacity to impact antigen levels. Redundancy in Ab effector systems may, therefore, not only exist to aid in protection against microbial challenge but also may serve as a mechanism to provide multiple avenues of protection against Ab-mediated injury to self. As CRs in particular are pleomorphic in function, removal of these receptors can result in a diverse range of phenotypes, only a part of which can be attributed to their role as CRs (68, 84). Therefore, understanding which receptor(s) may be involved in antigen removal, including the stage(s) in complement activation and degradation that may be responsible for this process, will be an important focus of future studies designed to determine how C3 facilitates antigen removal.

RBCs that express a single foreign and clinically relevant antigen not only aid in understanding key factors that may regulate RBC alloimmunization, but these models also provide a unique tool to understand the consequences of an Ab response in real time on target tissue. For many years, RBCs have provided an important substrate when seeking to study complement regulation and the consequences of Ab deposition on a host cell. Indeed, many of the seminal studies that describe key regulatory pathways of complement effector activity used RBCs as substrates when elucidating these pathways (94–98). The inability of RBCs to divide and synthesize new antigen eliminates many of the confounding variables that would make examination of alternative self-like substrates difficult to study (37, 99). Despite the use of RBCs for decades to study key regulators of complement effector pathways on the cell surface, opportunities to similarly take advantage of RBCs to study the impact of complement deposition on the development of an immune response have not been equally available. This largely reflects the fact that murine RBCs isolated from different strains of mice do not express antigenic determinants capable of inducing an immune response (35). As a result, examining an immune response to RBCs using intraspecies RBCs has not been possible. While injection of RBCs from other species, such as sheep RBCs, results in a robust immune response and has been used for many years to study host immunity (100–104), sheep and other foreign RBCs are rapidly cleared (29), which can result in an artificial acceleration of an immune response that can compromise direct comparison of host regulation of complement outcomes during Ab development. Furthermore, interspecies complement regulators are often less effective at regulating complement (105, 106), likewise reducing the ability to directly examine the outcome of complement regulation in an otherwise syngeneic system. Chemical attachment of antigen to the cell surface damages RBCs (107, 108), and also results in rapid clearance and the production of an inflammatory response that prevents isolation of a single antigenic determinant on an otherwise normal cell as a distinct variable when seeking to determine the outcome of target antigen exposure on self. Thus, models of RBC alloimmunization provide an opportunity to take advantage of all the unique features of RBC biology, including the ability of RBCs to circulate in a homogenous fashion that allows this population to be sampled over time, and thus examine in real time the impact of Ab formation on the RBC surface *in vivo*.

As in all experimental systems, limitations should be considered. Because our studies were performed in mice and not humans, our results may certainly inform our understanding of this clinical phenomenon observed in humans, but this is not a direct model of human RBC alloimmunization. Furthermore, the KEL RBC model is also unique given that the RBCs between recipient and donor mice are otherwise syngeneic, except for the expression of the KEL RBC antigen. This is unlike transfused human RBCs, where in the absence of alloantigen matching, recipients may receive RBCs that differ in multiple RBC antigens from the donor, potentially leading to RBC alloimmunization. However, this reductionist model in mice allows for studying the direct contribution of immunologic factors that may govern the alloantibody response to KEL, which would not be ethically feasible nor logistically possible to study in a detailed fashion in humans. This is in part due to the fact that while RBC alloimmunization occurs clinically as a consequence of therapeutic RBC transfusion, intentionally inducing RBC alloimmunization to antigens outside of RhD is not ethical, as it may put patients at risk for hemolytic transfusion reactions if emergent RBC transfusion is needed, as only ABO and RhD antigens are routinely considered in the emergent setting. Therefore, despite the differences between humans and mice, this model system provides a unique opportunity to gain insight into potential variables that may govern alloimmunization. More specifically, our data presented here may provide further understanding of how complement may regulate the alloantibody response to RBC antigens through modulating antigen on the RBC surface. This information may, therefore, provide the basis for future studies examining key determinants, such as complement, that may regulate an immune response following RBC transfusion.

Patients can develop an immune response following transfusion of various blood products, including the development of alloantibodies following exposure to distinct RBC alloantigens in the settings of therapeutic transfusion (8, 109). Similar to the outcome of this study, the development of Abs against polymorphic alloantigens on a RBC surface in patients often not only results in Ab formation, but also complement deposition (110, 111). However, unlike in mice of identical genetic backgrounds, the immune response in patients following RBC alloantigen exposure can vary significantly. While a variety of factors likely contribute to this phenomenon (112, 113), the results of this murine study suggest that variability in the levels and activity of complement proteins may impact this process. This is especially important when considering that many disease states that can become indications for RBC transfusion often exhibit significant variability in complement levels and complement deposition on circulating RBCs (114, 115). Thus, alterations in complement engagement following Ab deposition during initial Ab development may influence the magnitude and, therefore, the consequence of RBC alloantibody formation clinically. As a result, these studies not only provide fundamental insight into the role of complement in regulating Ab responses directed toward a target antigen expressed on a self-like RBC surface but also likely have clinical implications in the development of RBC alloantibodies, a process that significantly increases morbidity and mortality in patients who require repeat transfusion (4, 116).

## Ethics Statement

This study was carried out in accordance with the recommendations of the Emory Division of Animal Resources and the Institutional Animal Care and Use Committee. The protocol was approved by the Institutional Animal Care and Use Committee.

## Author Contributions

AM and SS designed experiments and wrote the manuscript. JL and JH contributed to study design. AM, CA, and SP performed experiments.

## Conflict of Interest Statement

The authors declare that the research was conducted in the absence of any commercial or financial relationships that could be construed as a potential conflict of interest.

## Supplementary Material

Figure S1 KEL or HOD expression is limited to red blood cells (RBCs). **(A)** RBCs, platelets, and white blood cells (WBCs) from KEL donor mice were assessed for KEL expression, along with the lineage-specific markers Ter119, CD41, and CD45, respectively. **(B)** RBCs, platelets, and WBCs from HOD donor mice were assessed for hen egg lysozyme (HEL) expression, along with the lineage-specific markers Ter119, CD41, and CD45, respectively.

Figure S2 Gating strategy for examination of red blood cells (RBCs). Ter119, CD41, and CD45 were assessed specifically on cells within the RBC gate.

Figure S3 HOD red blood cells (RBCs) and KEL RBCs induce similar anti-HOD and anti-KEL IgG subclass distribution following transfusion. B6 mice were transfused with either HOD or KEL RBCs, followed by evaluation of anti-KEL or anti-HOD IgG1 **(A)**, IgG2b **(B)**, IgG2c **(C)**, and IgG3 **(D)** in the serum on day 21 post-transfusion by flow cross-match. **(A–D)** ns = not significant. Means ± SD shown.

Figure S4 Anti-KEL antibodies fail to induce detectable C3b/iC3b deposition on circulating KEL red blood cells (RBCs) post-transfusion. C3b/iC3b deposited on circulating DiI-labeled KEL RBCs was measured at 10 min **(A)**, 4 h **(B)**, day 1 **(C)**, and day 3 **(D)** post-transfusion into KEL, B6, and C3 KO mice. **(A–D)** ns = not significant. Means ± SD shown.
