# Supplementary figures and images for: Complement Component 3 Negatively Regulates Antibody Response by Modulation of Red Blood Cell Antigen
Source: Front Immunol. 2018 Jun 11;9:676. doi: 10.3389/fimmu.2018.00676 (PMC6004516; doi:10.3389/fimmu.2018.00676)

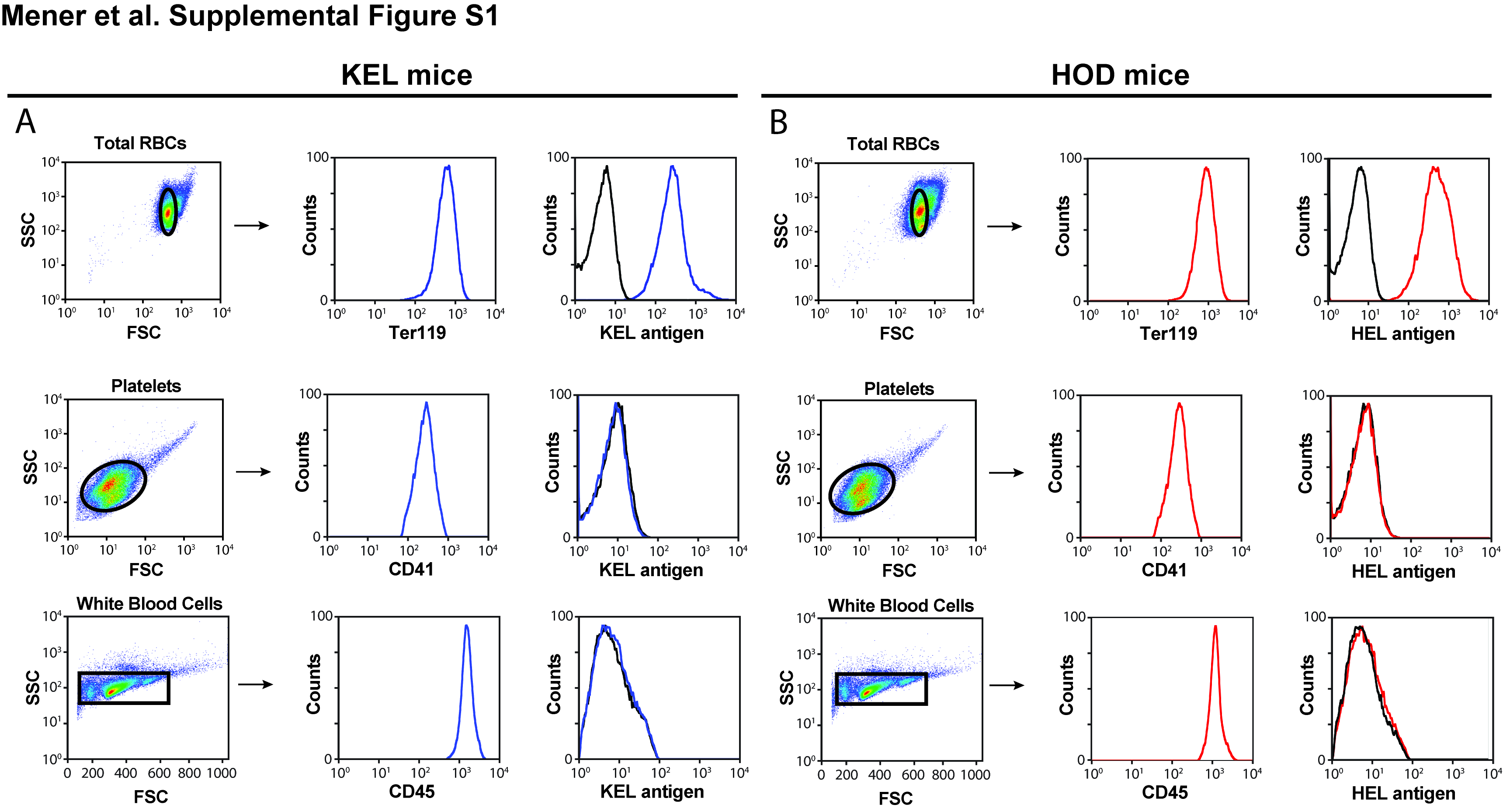

Supplement: Figure S1 — KEL or HOD expression is limited to red blood cells (RBCs). (A) RBCs, platelets, and white blood cells (WBCs) from KEL donor mice were assessed for KEL expression, along with the lineage-specific markers Ter119, CD41, and CD45, respectively. (B) RBCs, platelets, and WBCs from HOD donor mice were assessed for hen egg lysozyme (HEL) expression, along with the lineage-specific markers Ter119, CD41, and CD45, respectively. [file Image_1.tif]

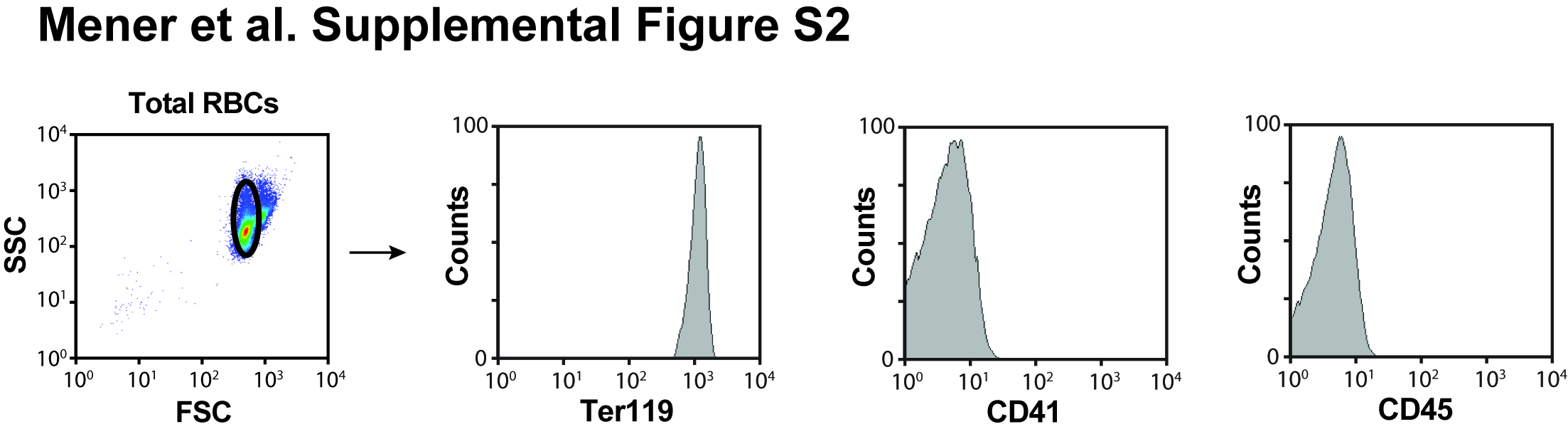

Supplement: Figure S2 — Gating strategy for examination of red blood cells (RBCs). Ter119, CD41, and CD45 were assessed specifically on cells within the RBC gate. [file Image_2.tif]

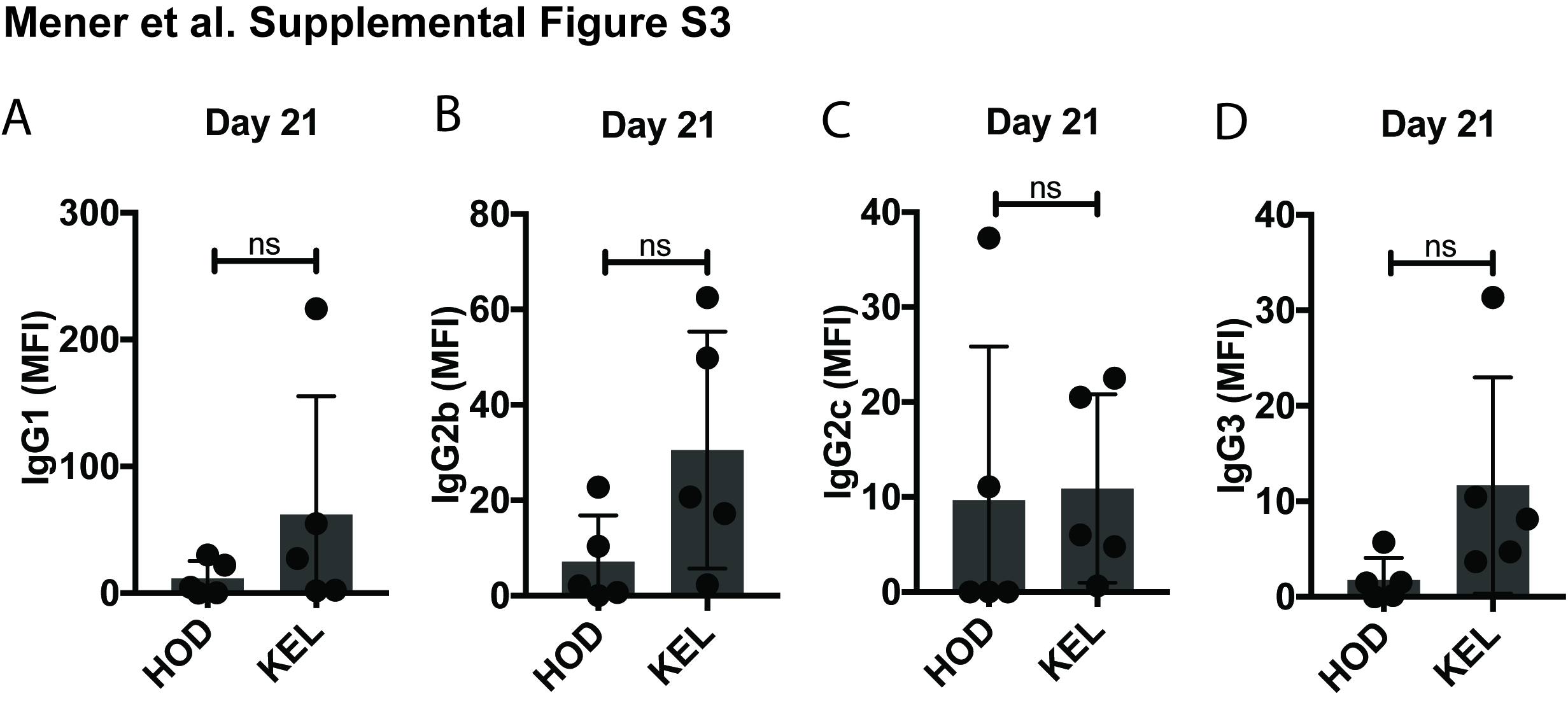

Supplement: Figure S3 — HOD red blood cells (RBCs) and KEL RBCs induce similar anti-HOD and anti-KEL IgG subclass distribution following transfusion. B6 mice were transfused with either HOD or KEL RBCs, followed by evaluation of anti-KEL or anti-HOD IgG1 (A), IgG2b (B), IgG2c (C), and IgG3 (D) in the serum on day 21 post-transfusion by flow cross-match. (A–D) ns = not significant. Means ± SD shown. [file Image_3.tif]

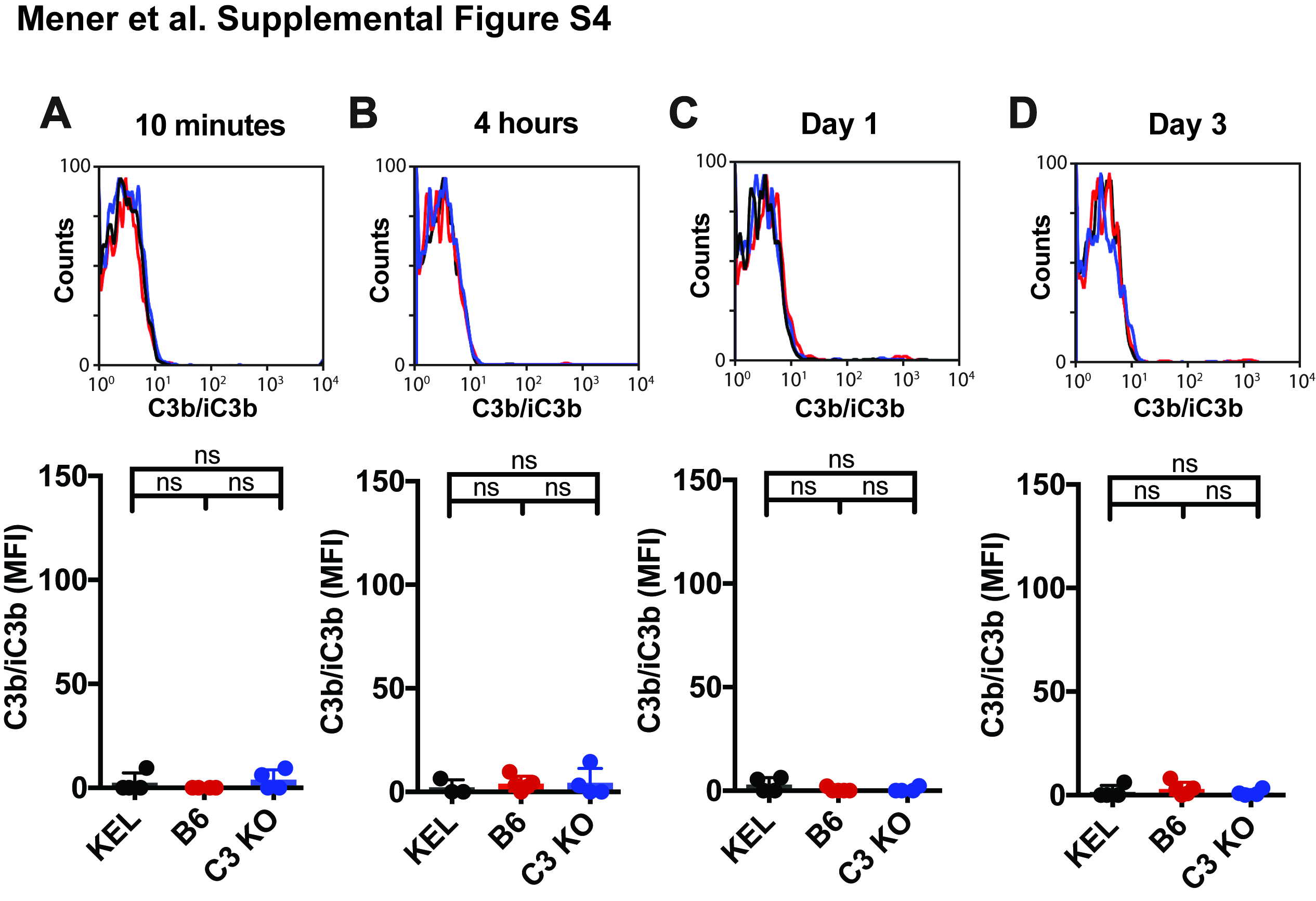

Supplement: Figure S4 — Anti-KEL antibodies fail to induce detectable C3b/iC3b deposition on circulating KEL red blood cells (RBCs) post-transfusion. C3b/iC3b deposited on circulating DiI-labeled KEL RBCs was measured at 10 min (A), 4 h (B), day 1 (C), and day 3 (D) post-transfusion into KEL, B6, and C3 KO mice. (A–D) ns = not significant. Means ± SD shown. [file Image_4.tif]
